# The Role of Information Technology Mindfulness in the Postadoption Stage of Using Personal Health Devices: Cross-Sectional Questionnaire Study in Mobile Health

**DOI:** 10.2196/18122

**Published:** 2020-10-05

**Authors:** Pouyan Esmaeilzadeh

**Affiliations:** 1 Department of Information Systems and Business Analytics Florida International University Miami, FL United States

**Keywords:** IT identity, IT mindfulness, personal health devices, perceived health status, post-adoption behaviors, mHealth, mobile phone

## Abstract

**Background:**

Although personal health devices (for example, smartwatches, fitness trackers and intelligent bracelets) offer great potential to monitor personal fitness and health parameters, many users discontinue using them after a few months. Thus, it is critical to study the postadoption behaviors of current users to enhance their engagement with personal health devices and use behaviors. However, there is little empirical research on the factors affecting users’ engagement in beneficial use behaviors. Mindfulness and identity are not new topics, but the applications of these concepts in the field of information systems are emerging themes. Information technology (IT) mindfulness has been conceptualized in previous studies; however, little is known about the antecedents and consequences of IT mindfulness in the mobile health (mHealth) context.

**Objective:**

The main aim of this study is to explore both IT identity and IT mindfulness to develop a new ground for research in the domain of mHealth postadoption. Thus, we aim to explain why users should be fully mindful of their engagement with PHDs and what could be the consequences and implications.

**Methods:**

This study proposes that IT mindfulness can play an important role in improving the use behaviors of users. Through a web-based survey with 450 current users of a personal health device, this paper tests the relationship between IT identity and IT mindfulness in the postadoption stage of using personal health devices.

**Results:**

We found that IT identity significantly shapes IT mindfulness associated with PHDs. Moreover, the IT identity–IT mindfulness relationship is negatively moderated by individuals’ perceived health status (*P*=.003). Finally, the results of this study show that IT mindfulness can significantly predict automatic use behaviors (eg, continued intention to use), active use behaviors (eg, feature use and enhanced use behaviors), and commitment behaviors in using personal health devices (eg, positive word-of-mouth intention).

**Conclusions:**

The findings of this study provide implications for both research and practice. This study can contribute to our current understanding of IT mindfulness by developing and empirically testing a research model that explains the determinants and outcomes of the IT mindfulness construct in the context of personal health devices. The results imply that IT mindfulness significantly helps individuals express their alertness, awareness, openness, and orientation in the present in their postadoption interactions with smart devices used for health care purposes. Finally, our findings may assist practitioners and IT developers in designing mindfulness-supporting PHDs. Owing to the impact of IT mindfulness on postadoption behaviors, its 4 dimensions could be used for developing PHD technologies. Moreover, PHD developers may need to direct their efforts toward increasing IT mindfulness by reinforcing IT identity to serve and retain a wide range of target users.

## Introduction

### Background

Mobile health (mHealth) has tremendously changed how people are involved in performing different roles in their social relations [1]. In recent years, the use of personal health devices (PHDs) as platforms for monitoring and controlling health conditions has grown significantly. Many manufacturers (eg, Samsung, Jawbone, and Fitbit) have developed smartwatches and intelligent bracelets to help individuals fulfill their health-related goals. Use of information technology (IT) allows individuals to feel competent and accepted in social networks of relationships in which their roles are defined based on cultural expectations and norms [2]. For instance, PHDs can enable individuals to monitor their health status, manage chronic diseases, control fitness as well as wellness, and view personal health information in real time [3]. In this study, PHDs are defined as smart devices used for monitoring and controlling health status, not for providing diagnoses and medical treatment remotely. People may continuously engage with their PHDs to monitor their health status or check their health care information [4]. Thus, the use of PHDs is beneficial to people because these devices provide them with additional resources in managing personal health care, which is highly important to them. These devices can verify identity by providing broad applications across a wide range of technology networks and social contexts. These smart devices can be integrated into an individual’s sense of self, as one may spend a significant portion of their time interacting with the devices as a repeated behavior. This is consistent with the findings of Reychav et al [5] that repeated behaviors can directly contribute to identity construction.

Technology can be manifested in different ways, for instance, technology interface or what the technology can afford. This study does not focus on the technology interface, but we examine technology in terms of how people use the platform to identify the underlying application, purpose, and function. Individuals may interact with many technologies daily but may consider only certain IT devices as an inherent part of themselves, which can influence their behavioral choices [6]. A PHD (eg, a wearable smart device or an intelligent bracelet) is a consumer IT, which could become part of people’s identities because of its everyday use for self-monitoring and self-management purposes [7]. The adoption and the use of patient self-management tools are consistent with the notion of patient-centered eHealth apps, which revolves around patients as pivotal actors in the health care ecosystem [8]. However, recent studies highlight that many users discontinue using PHDs after only a few months [9]. Little is known about the factors that motivate users to actively use such electronic devices and explore more features and functions for additional health-related tasks [10]. Thus, further research is required to investigate individuals’ beliefs and behaviors during the postadoption of PHDs to uncover users’ requirements and preferences for eHealth services and to propose suggestions for the construction of effective smart devices, interactive mHealth apps, and efficient eHealth systems.

To fill this gap, we use the concept of IT mindfulness as a theoretical foundation to delineate how PHD users perform postadoption behaviors. Mindfulness is a multidimensional concept that explains constant examinations, expectation refinement, recognition of new changes, and exploration of novel aspects of a phenomenon [11]. According to Weick and Sutcliffe [12], mindfulness refers to focusing on the present, paying attention to details, considering alternative perspectives, and willingness to investigate for understanding system failures. In line with a study by Ndubisi [13], people who are mindful are very sensitive to different contexts and have the ability to continuously create new categories in awareness and interpretation of the world. In contrast, individuals who engage in mindless behaviors have a premature commitment to rigid beliefs and preexisting categories [14].

Mindfulness promotes being in a watchful and vigilant state of mind [14]. A study by Sun et al [15] suggests that IT mindfulness may influence technology adoption decisions and continuance. Their results indicate that mindful users continuously compare an IT with existing technologies to be aware of its uniqueness and make more rational postadoption decisions in the light of task-technology fit. A mindful process of experimenting with IT can articulate how individuals extract value from technologies [16]. IT mindfulness may reduce the addictive use of technology and help individuals feel comfortable with their use of IT systems. Previous research indicates that domain-specific mindfulness (eg, in situations related to personal health) could predict individual behaviors [17]. IT mindfulness, as an IT-specific trait, influences users’ behaviors with a given technology [18]. As IT mindfulness is considered a dynamic IT-specific trait, this class of trait likely influences technology-related behaviors such as user acceptance of technology and postadoption use behaviors.

Previous studies provide significant evidence to show that IT mindfulness shapes future behavior by explaining how awareness and flexibility of users can alter their future interactions with the IT system [19]. People with high levels of mindfulness tend to continually monitor the current situation to find new ways of using IT, which helps them complete their tasks [20]. Beliefs and behaviors are affected by personality traits (at different hierarchical levels). Previous studies indicate that dynamic and context-specific traits (rather than broad traits) are more likely to change specific behaviors [21]. However, little is known about the effects of information system (IS)–specific traits (such as IT mindfulness) on IT-specific beliefs and use behaviors. Moreover, there is an increase in studying the causes and consequences of mindfulness in different research areas [22]. However, most of these studies are conducted in the organizational context and are considered organizational factors and interventions [22]. Another stream of research focuses on mindfulness in a collective context, such as a group setting, rather than examining mindfulness at the individual level [23]. Thus, more studies are required to examine the antecedents and resultant effects of IT mindfulness at the individual level in the context of using consumer IT.

### Objectives

The main goal of this study is to highlight the ways in which the application of the IT mindfulness concept in studies of mHealth device design and use can contribute to the realization of its antecedents and outcomes in the PHD context. We propose that IT mindfulness may provide a possible basis for answering questions about how individuals can hope to efficiently use smart devices to achieve reliable health-related results. We argue that positive use behaviors are possible when a mindful approach permeates an individual user. In doing so, we develop a research model by drawing on the recent appearance of the concept of mindfulness in the IS literature and adapting it for application to postadoption of PHDs. In brief, this study addresses the following research questions:

1. How does IT mindfulness influence individuals’ postadoption interactions with PHDs?
2. What are the antecedents of IT mindfulness in the context of PHDs?
3. How do health factors (ie, perceived health status) moderate the influencing chain from IT identity to IT mindfulness?

### Literature Review

#### IT Mindfulness

Mindfulness is a psychological trait that has roots in the cognitive abilities of the individual [20]. Mindfulness has been used in IS studies with different themes, such as IT innovation, IT management, IT use, and outcomes [24]. Swanson and Ramiller [20] suggest that the concept of mindfulness can be incorporated into the adoption, implementation, and assimilation of an IT innovation. As a result, IT mindfulness arises when people are working with IT. When people are aware of IT capabilities and open to its various functions, they elevate their mental mindset to become mindful of value-adding applications of IT. People with cultivated IT mindfulness are likely to focus on the present IT functionalities, search for more details about its applications, explore more uses of IT, and examine IT features [25]. When a person mindfully accepts a technology, they are aware of the given technology, its functions, and their needs. Thus, they are more likely to search for more details and information about the technology and the acceptance decision and possible implications [26].

Sternberg [27] describes the concept of mindfulness as (1) alertness to distinction, (2) awareness of multiple perspectives, (3) openness to novelty, and (4) orientation in the present. Consistent with previous studies, we consider IT mindfulness as a second-order construct that consists of 4 reflective dimensions [25,28]. The first first-order dimension is alertness to distinction, which refers to the ability to define, appreciate, and make judgments about IT applications and their potential. This factor helps individuals identify the differences between the old and new features of an IT application and seek new ways to use the system. The second dimension is awareness of multiple perspectives, which helps individuals analyze IT system applications and features from different or even opposing viewpoints. This dialectical thinking may lead to innovative solutions to IT-related problems. The third dimension is the openness to novelty, which involves curiosity and flexibility in a user’s interactions with an IT system’s features and applications. This factor is instrumental in cultivating in a user the ability to reason about new types of stimuli, consider a large number of IT applications, and explore fewer familiar features. The last factor is an orientation in the present, which manifests the degree to which an individual pays more of his/her attention to his/her current situation instead of envisioning future possibilities or concentrating on past events. This factor may increase people’s sensitivity to the immediate context and adapt their responses and system used to the current situation. As a second-order construct with reflective dimensions, IT mindfulness requires capturing all 4 dimensions. Higher levels of IT mindfulness will lead to greater levels of the 4 dimensions. We cannot assume that a change in IT mindfulness will lead to the same amount of change across the 4 dimensions [29]. Although the indicators of dimensions may covary, each dimension has a separate conceptual foundation [30].

#### IT Identity

Prior researchers have studied the topic of IT and identity and their relationship based on different approaches. Carter and Grover [31] conceptualize IT identity using theories on social structures and self-concept to describe how people categorize themselves in relation to an IT object. They define IT identity as the extent to which the use of IT is saliently related to who people think they are (self-identification). IT can change individuals’ self-perceptions by recognizing their original selves in using the capabilities and resources offered by the IT device. For instance, in the presence of IT, people may feel empowered, productive, autonomous, and accessible. According to Carter et al [32], adults’ interactions with their mobile phones lead to enhanced perceptions of empowerment, self-authenticity, and autonomy. In the context of PHDs, the self-concept and personal resources of users can be expanded by the capabilities provided by the devices. For example, wearable smart devices can be used to reduce time and place constraints in controlling fitness and wellness, measuring different physical changes, and handling emergency cases. These functionalities may enhance individuals’ original self-perceptions and may make them feel independent, empowered, and smart.

Previous studies on IT identity suggest that the construct of IT identity is expressed through 3 first-order factors: relatedness, dependence, and emotional responses of individuals in relation to IT [31]. Relatedness refers to a sense of connection felt when interacting with an IT device. For instance, a strong feeling of connection with an IT device can turn individuals’ perceptions about the self to what they can do with the IT [33]. Emotional energy indicates the levels of emotional attachment, enthusiasm, and confidence that an individual attributes to an IT when thinking about his/her interaction with it. Long-term interactions with an IT system can raise individuals’ levels of emotion and confidence in relation to the IT experienced and help them to be more spontaneous with the IT [34]. Dependence explains the extent to which people rely on IT to represent their self-perceptions. For instance, people rely on digital forms of communication to manage their interactions and relationships with others to satisfy social expectations [35].

### Hypothesis Development

#### IT Identity and IT Mindfulness

Using the hierarchical structure of personality traits, broader IS-specific traits (ie, qualities or characteristics belonging to a user that developed and were enacted because of using a particular technology) can influence narrower dynamic traits [36]. For instance, previous studies suggest that context-specific traits are closely related to dynamic IT-specific traits (such as IT mindfulness) [22]. Owing to the malleability of this trait, personal interactions with technology may nurture IT mindfulness and encourage users to gain more value from PHDs. In line with this rationale and based on the scope of IT identity and IT mindfulness, we propose that IT identity will help shape IT mindfulness. IT identity is a broad trait because it is the first step that users will attach dependence, emotion, and relatedness to a technology. This IS-specific trait may encourage users to gather more information about the applications and functions of that technology, analyze its applications and features, and seek new ways to use the system.

Users who hold a higher level of IT identity associated with a PHD will be generally dependent on their device, have an overall sense of connection when interacting with it, and develop emotional energy toward using it. IT identity can change individuals’ self-perceptions by recognizing their original selves in using the capabilities and resources offered by the IT device. For instance, in the presence of IT, people may feel empowered, productive, autonomous, and accessible. These feelings function broadly because of the sense of independence, and the feeling of being smart or productive can nourish their overall self-perceptions [37]. The self-concept (which is influenced by IT) can then reinforce the users’ readiness to increase their understanding of the functions of a specific IT, vigilance to its differences, sensitivity to its current task context, and curiosity to its features. Therefore, using the hierarchical structure of personality traits, we contend that IT identity may be placed at a higher hierarchical level, and it could be an antecedent of shaping IT mindfulness.

A PHD user with a strong IT identity is highly dependent on his/her device [38]. This leads the IT identity holder to be greatly alert to distinction and such a user tends to identify new ways to accomplish health-related tasks by using his/her device (alert to the distinction). A higher level of IT identity makes users have a strong feeling of connection with a PHD. This makes them more willing to get involved when using their PHD and to keep a constant eye on the big picture to differentiate between usage contexts (orientation to the present) [25]. Users with strong IT identity develop higher levels of emotional attachment, enthusiasm, and confidence that they attribute to a PHD when thinking about their interaction with it [31]. This feeling enables users to be open to new ways of using a PHD and be more eager to learn new ways of using it (awareness of multiple perspectives). IT identity holders rely highly on a PHD to manage their health metrics to satisfy their personal health-related expectations. This self-concept encourages users to explore new potential or features within their device (openness to novelty) [18].

Interacting with an IT device that contributes to self-identification can be a considerable opportunity for IT users. When a person considers a PHD as an integral part of the self, he/she may quickly recognize the capabilities and potential of a PHD, the differences between its features, and the utility of its new/updated features. A strong IT identity can motivate users to attribute higher levels of emotional attachment, enthusiasm, and confidence in his/her medical device and, in turn, become involved in the PHD usage through constant adoption of its new features. An IT identity holder may seek more information, increase his/her knowledge about its features, and hold diverse perspectives toward the potential usage of a PHD and become inspired to develop innovative solutions using these different perspectives. A higher level of IT identity may impart more confidence to the users to rely on PHDs to explore new features within a PHD and be flexible to new features of their PHD. Thus, we posit that IT identity affects the degree to which users develop mindfulness in using their PHDs.

Our first hypothesis is as follows:

- H1: IT identity positively influences the IT mindfulness of PHD users.

#### The Moderating Role of Perceived Health Status

According to Bansal and Gefen [39], individuals’ characteristics (such as current health status) significantly affect the way they analyze the utility of using IT. Perceived health status is a common health factor that highlights overall individual health [40]. Previous research highlights the effects of perceived health status in different contexts. For instance, personal health status evokes privacy concerns related to health information disclosure and the tools used to share such information [41]. Previous research highlights that mindfulness is positively related to higher levels of well-being [42]. Most studies on mindfulness suggest that mindfulness will lead to pleasant psychological effects. For instance, acting mindfully is closely related to improved psychological health [43]. Brown and Ryan [44] reported that mindfulness could improve self-esteem and optimism and reduce anxiety. Cash and Whittingham [45] demonstrated a negative relationship between mindfulness and depression. A study by Raes et al [46] also showed that mindfulness positively influences cognitive reactivity. A review study contends that mindfulness-oriented interventions are significantly associated with positive psychological effects, such as better subjective well-being, reduced psychological distress, and improved behavioral regulation [47].

However, little is known about whether the overall evaluation of a person about his/her health status can affect the development of IT mindfulness and IT identity. We argue that perceived health status may change the way people think about themselves, their capabilities, and the world around them. Unhealthy individuals (eg, with chronic diseases) perceive more strain because of the presence of physical/mental infirmity, and this health condition makes them more anxious and vulnerable to the digital devices surrounding them. People in good health tend to assume less severe demands on their strength or abilities, perceive more control, and experience less mental/emotional strain [48].

Therefore, the impact of IT identity on IT mindfulness in a given situation may depend on a user’s perception of his/her health status. We expect that the IT identity–IT mindfulness relationship may vary depending on the current health status of users. In doing so, the overall sense of well-being can influence the level of PHD users’ attachment, reliance, and dependence on their devices to perform health-related tasks. In turn, it may induce changes in the degree of awareness of various features, attention to the present moment experience, alertness to differences, and openness to new information. On the basis of the moderating effect of perceived health status, we posit that people with high IT identity levels may not always tend to remain high in IT mindfulness, and their perceptions about their well-being may change the strength and direction of this relationship. For instance, in the PHD context, an IT identity holder may think of using the body temperature function, high heart rate notifications, or insulin delivery features to monitor their health status rather than making an appointment with a physician. By doing so, he/she will enact his/her identity as a competent person who can leverage a PHD to keep records of his/her health conditions. However, current studies cannot answer whether this high IT identity will always be translated into high IT mindfulness. Thus, we propose that the IT identity–IT mindfulness linkage could be moderated (augmented or attenuated) by perceived health status.

Our second hypothesis is as follows:

- H2: Perceived health status moderates the relationship between IT identity and IT mindfulness in using PHDs.

#### IT Mindfulness and Postadoption Behaviors

In line with previous research [44], mindfulness is a state of consciousness that facilitates the fulfillment of basic psychological needs. In turn, mindfulness is a good predictor of self-regulated behaviors. Consequently, IT mindfulness as an IT-specific trait can be used to study IT-related beliefs and behaviors. Previous studies examined the impact of mindfulness on the formation of people’s beliefs about using technology [26]. For instance, IT mindfulness strongly shapes the perception of technostress [22]. Previous research demonstrates the significant effects of mindfulness on innovating with IT [20]. Thatcher et al [25] demonstrated that IT mindfulness significantly influences deep structure usage and attempts at innovation.

We also tested the relationships between IT mindfulness and important systems use constructs. In this study, the postadoption system use behavior is represented by feature use, enhanced use, continued intention, and positive word-of-mouth (WOM) intention. Active system use refers to a situation in which people ponder the system and knowingly modify how to use it [49]. In previous studies, automatic system use usually implies habitual behaviors where people use the system unconsciously without thoughtful assessments and focused analyses related to their use [50]. However, in this research, automated system use reflects continued intention to use a PHD based on a mindful consideration of alternatives, not the addictive use of technology. Therefore, more active system use is denoted by feature use as well as enhanced use behaviors, and automated system use is represented by continued intention to use.

According to Sun et al [15], mindful IT users are more willing to use different features of IT. When being mindful, the user is more likely to actively explore and discover additional useful features and functions of a technology [26]. Individuals who are mindfully engaged in a health-related task using a PHD are more motivated to explore a wide range of perspectives. Engagement in feature use behaviors requires sharp user alertness and dynamic awareness of how the use of various features and applications can contribute to task completion [51]. Involvement in enhanced use entails users to explore previously unused features of a PHD to use it for performing additional tasks [52]. Mindfulness helps people scan the context for interpreting the context-relevant information of all conditions [14]. People with higher levels of mindfulness tend to know their context as well as their ability, and they are more open to deliberately search for new features to complete further health-related tasks.

Hypotheses 3 and 4 are as follows:

- H3: IT mindfulness positively influences feature use behavior of PHD users.
- H4: IT mindfulness positively influences enhanced use behavior of PHD users.

Continued intention implies the extent to which people are likely to use familiar technology in the future. Mindful thinking increases people’s willingness to process information and continue to use the features of IT in an alert and open way [28]. Mindful PHD users are more likely to have a sense of control when using this technology because they clearly know what they can and cannot do with their smart devices. According to Wong et al [53], mindfulness indicates being open to new information about the technology at hand, being aware of various perspectives, and being involved in the continuous creation of options. Mindful people are more likely to be receptive to new information and compare the technology being used with others [26]. In addition, mindful users are more likely to recognize all the consequences of their decisions (eg, both the pros and cons). Given that they have more information about the system, as long as the current technology seems beneficial, mindful users are likely to continue using the same system compared with individuals who use the system mindlessly.

Hypothesis 5 is as follows:

- H5: IT mindfulness positively influences continued intention to use PHD.

Mindfulness reinforces learning from interpreting related outcomes [24]. When acting in a mindful way, one pays more attention to every detail of IT applications at hand and becomes sensitive to the context. By exploring new aspects of IT and understanding its capabilities and potentials, the user will be open to resolving any challenging situation to accomplish his/her tasks more effectively. Thus, IT mindfulness may positively influence user satisfaction with the technology used to accomplish his/her tasks [18]. Consistent with Fiol and O'Connor [14], mindful users actively analyze how a PHD fits their own contexts and needs rather than blindly follow others in using it. As a result, they may be more inclined to describe the features and functions of PHDs to others and encourage them to use these smart devices to fulfill their health-related needs. Achieving a fit between the technology and the task may encourage users to be committed to PHDs and become more likely to make positive comments about the mobile system they are using. Thus, we hypothesize that IT mindfulness can enhance positive postadoption behaviors (ie, active use behaviors, continued use behaviors, and positive WOM intention of PHD users).

Hypothesis 6 is as follows:

- H6: IT mindfulness positively influences the positive WOM intention of PHD users.

### Research Model

**Figure 1:**
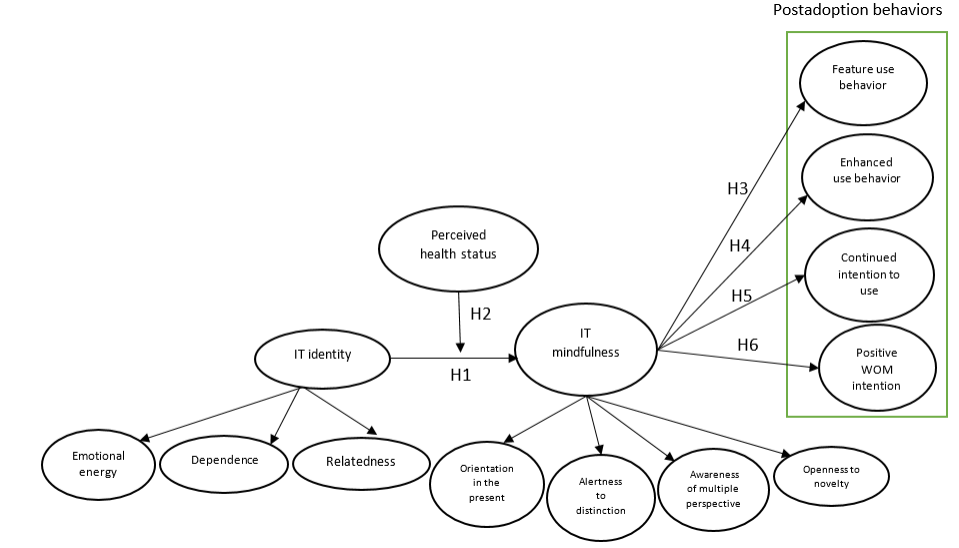
Research model. IT: information technology; WOM: word-of-mouth.

We bring IT identity and IT mindlessness together in a theoretical synthesis in which these concepts are seen to interact in ways that help shape the postadoption behaviors of PHD users. The research model indicates that IT identity with PHDs can build IT mindfulness and, in turn, will lead to positive postadoption use behaviors. However, according to Carter and Grover [31], individuals do not always attempt to use IT to exhibit who they are to others even when the IT is advantageous to them. Moreover, previous studies suggest that mindfulness skills are significantly related to aspects of health status [54]. Thus, we suggest that the verification of IT identity–IT mindfulness linkage in relation to the use of PHDs may depend on the health status of people. As the probability of IT identity and IT mindfulness may be evoked by perceived health status, the relationships between IT identity and IT mindfulness can be moderated by this health factor. Figure 1 shows the proposed research model.

## Methods

### Definition of Variables

The final measure items (ie, all the items included in the survey) are listed in Multimedia Appendix 1. Table 1 provides the definitions of the constructs used in this study.

**Table 1:** Operationalization of variables.

Construct	Construct definition	Source
IT^a^ identity	The degree to which the use of an IT (ie, a PHD^b^) is meaningfully related to who people think they are (self-identification).	Carter and Grover [31]
IT mindfulness	The degree to which a user is involved in the present context, alert of details, aware of other potential uses, and open to investigating IT (ie, a PHD) features and failures.	Thatcher et al [25]
Perceived health status	The extent to which an individual believes that the overall status of his/her health and wellness is good.	Bansal and Gefen [39]
Positive word-of-mouth intention	The degree to which a user shares a positive assessment of his/her experience with a PHD with others.	Maxham III [55]
Continued intention to use	The degree to which a user feels he or she will keep using a PHD.	Bhattacherjee [56]
Feature use behavior	The extent to which an individual uses various features and functions of a PHD in different situations.	Lucas Jr and Spitler [57]
Enhanced use behavior	The extent to which an individual explores previously unused features of a PHD to use it for performing additional tasks.	Bagayogo et al [52]

**^a^**IT: information technology.

^b^PHD: personal health device.

### Measurement of Variables

This study drew on the existing literature to measure the constructs included in the model, and minor changes were made to the instrument to fit the PHD context. Consistent with prior studies, we consider IT identity as a second-order construct with 3 reflective factors [58]. The rationale behind this measurement is that IT identity is reflective of the 3 dimensions as well as the expected interactions among them. Therefore, all these dimensions can reflect the same theme and may covary. To measure the 3 interrelated dimensions of IT identity (ie, relatedness, emotional energy, and dependence), we adapted the items reported in Carter and Grover [31]. According to Kayhan [59], reflective modeling is a better option than formative when first-order factors are expected to interact, correlate, or share a common theme. Thus, a set of interrelationships among these factors is an essential component for measuring IT identity. For instance, dependence, which defines a person’s sense of reliance on a PHD, may be related to the emotional energy dimension that describes the feelings of attachment in relation to the device.

In line with a study by Langer and Ngnoumen [60], IT mindfulness is also modeled as a second-order construct composed of 4 reflective first-order dimensions: (1) alertness to distinction, (2) awareness of multiple perspectives, (3) openness to novelty, and (4) orientation in the present. Thus, each dimension has a distinct conceptual foundation, and the items of these 4 dimensions may covary and become interchangeable. On the basis of reflective modeling, individuals with higher levels of IT mindfulness are more likely to exhibit higher levels of alertness to distinction, awareness of multiple perspectives, openness to novelty, and orientation in the present. A change in the IT mindfulness construct may not lead to the same amount of change across all 4 dimensions [29]. Thus, in this study, IT mindfulness is operationalized as a construct that requires capturing all 4 dimensions. We adapted reflective items from a study by Thatcher et al [25] to measure the 4 dimensions of IT mindfulness.

The outcome variables studied in this research are feature use behavior, enhanced use behavior, continued intention to use, and positive WOM. These outcome variables are considered as different types of postadoption behaviors. Items reflecting feature use behaviors were adapted from a study by Lucas and Spitler [57], and items measuring enhanced use behaviors were adapted from a study by Bagayogo et al [52]. Items measuring continued intention to use were adapted from studies by Venkatesh and Goyal [61] and Bhattacherjee [56]. We adapted the items reported in a study by Hoehle and Venkatesh [62] to measure positive WOM intention. Finally, items measuring perceived health status were adapted from the scale developed by Bansal and Gefen [39].

### Participant Recruitment

Data were collected in October 2019 from Amazon’s Mechanical Turk (MTurk) to obtain a representative group of subjects in the United States. As PHDs may not be considered as a routine technology for many individuals, to obtain more robust, reliable, and applicable findings, we specified 2 more qualifications that individuals had to meet to participate in the survey. First, we defined a screening question to only include individuals who have been using a PHD. We attempted to distinguish between PHDs (hardware device) and mHealth apps (software app) and only include users of a mobile smart device. The logic behind this screening is that we defined IT as a unit of technology (hardware device and software app). Accordingly, the dimensions of IT mindfulness and IT identity can be properly measured and examined, resulting from interactions with devices (as a unit) and not through interaction with application environments or software apps. For example, participants of this study were users of any PHDs (such as wearable smart devices, wearable activity monitors, and intelligent bracelets). When individuals use a PHD, they are exposed to their features and characteristics. Therefore, the likelihood that they become more familiar with its functions and mechanisms is greater, and IT mindfulness as well as IT identity are more likely to be enacted. Thus, we ensured that the participants had used PHDs when they took part in this study. The incentive for participation was a monetary reward (US $3). At the beginning of the web-based survey, a detailed description of PHDs was provided to ensure that respondents completely comprehended the context and purpose of the study. In total, 462 individuals attempted the survey.

Second, as mentioned in previous studies, one general concern in data collection is a potential lack of attention and random responses [63]. Consistent with other studies, we used some attention trap questions to prevent and identify careless, hurried, or haphazard answers [64]. On the basis of answers to these attention-trap questions, 12 responses were dropped. This ratio is similar to those reported in previous studies that used MTurk for data collection [65]. Thus, concerns that web-based respondents might reply randomly or haphazardly to complete the survey quickly were alleviated. After excluding responses that failed the response quality questions, the final sample consisted of 450 usable and valid questionnaires. We also used Mplus to assess the power of the analysis and determine the sample size [66]. Given the number of observed and latent variables in the model, the anticipated effect size (0.3), the desired probability (0.8), and statistical power levels (α=.05 and power β=.95), the minimum sample size for the model structure is 400. Therefore, this study was adequately powered, as 450 respondents could be sufficient to reduce possible sampling errors and minimize type 2 errors. This is consistent with both the ratio of indicators to latent variables approach and the function of minimum effect, power, and significance suggested by Westland [67].

## Results

### Descriptive Statistics

Table 2 depicts the respondents’ characteristics. The demographic characteristics show that most respondents were female (270/450, 60.0%), White (301/450, 66.9%), with a full-time job (311/450, 69.1%), and had a bachelor’s degree (257/450, 57.1%). Approximately 69.7% (314/450) of respondents were aged between 20 and 39 years, and approximately half had an annual household income between US $25,000 and US $74,999. Regarding experience, frequency, and length of use, the results imply that the respondents of this study were familiar with a PHD. All participants had used a PHD before, and most (276/450, 60.8%) rated themselves as either very experienced or extremely experienced with an mHealth device. Overall, 62.0% (279/450) of respondents used PHD daily, and approximately 52.2% (235/450) used PHD for more than a year. Finally, respondents were asked to indicate the type of their PHDs and the purpose of using them. Fitbit (176/450, 39.1%), Apple Watch (90/450, 20.0%), and Samsung Galaxy Fit (81/450, 18.0%) were the top 3 PHDs used by respondents. Controlling fitness and diet (203/450, 45.1%) followed by monitoring blood pressure and checking the cholesterol level (122/450, 27.1%) received the highest percentage of responses regarding the purpose of use.

**Table 2:** Sample characteristics (N=450).

Variables		Value, n (%)
**Gender**		
	Male	180 (40.0)
	Female	270 (60.0)
**Age (years)**		
	<20	5 (1.1)
	20-29	157 (34.9)
	30-39	157 (34.9)
	40-49	82 (18.3)
	50-59	36 (8.0)
	≥60	13 (2.9)
**Annual household income (US $)**		
	<25,000	72 (16.0)
	25,000-49,999	115 (25.5)
	50,000-74,999	112 (24.8)
	75,000-99,999	75 (16.6)
	≥100,000	76 (16.8)
**Education**		
	Less than high school	22 (4.9)
	High school graduate	45 (10.0)
	Some college	77 (17.1)
	2-year degree	35 (7.7)
	Bachelor’s degree	257 (57.1)
	Graduate degree	14 (3.1)
**Employment status**		
	Employed full time	311 (69.1)
	Employed part time	73 (16.3)
	Unemployed	33 (7.4)
	Retired	10 (2.2)
	Student	23 (5.1)
**Race and ethnicity**		
	White	301 (66.9)
	African American	51 (11.4)
	Asian	27 (6.0)
	Hispanic	66 (14.6)
	Mixed	5 (1.1)
**Experience with mobile devices (eg, phone, tablets)**		
	Slightly experienced	8 (1.7)
	Moderately experienced	77 (17.1)
	Very experienced	145 (32.3)
	Extremely experienced	220 (48.9)
**Experience with PHDs^a^**		
	Slightly experienced	40 (8.9)
	Moderately experienced	134 (29.7)
	Very experienced	172 (38.3)
	Extremely experienced	104 (23.1)
**Frequency of use**		
	Rarely	8 (1.7)
	Monthly	44 (9.7)
	Weekly	119 (26.6)
	Daily	279 (62.0)
**Length of use**		
	<6 months	84 (18.6)
	6 months to 1 year	131 (29.1)
	1-2 years	110 (24.6)
	>2 years	125 (27.7)
**PHDs used by participants**		
	Fitbit	176 (39.1)
	Apple Watch	90 (20.0)
	Samsung Galaxy Fit	81 (18.0)
	FitTech Smart Watches	58 (12.8)
	Garmin Fitness Watches	27 (6.0)
	Other Smart Fitness Trackers	18 (3.9)
**Purpose of use**		
	Controlling fitness and diet	203 (45.1)
	Monitoring blood pressure and checking the cholesterol level	122 (27.1)
	Controlling or quitting smoking	54 (12.0)
	Monitoring chronic diseases (eg, diabetes and heart disease)	45 (10.0)
	Controlling depression or anxiety	26 (5.8)

^a^PHD: personal health device.

### Instrument Validations

Before data were statistically analyzed, normality was evaluated, as this is important for the distribution of data to exhibit this trait, to facilitate unbiased and consistent models [68]. Thus, all the constructs used in the model were scrutinized against the normality assumptions. An examination of the skewness and kurtosis of the constructs showed a skewness range from 0.045 to 1.164 and a kurtosis range from 0.017 to 1.531. On the basis of these findings, all the values fall within the prescribed limit and maximum acceptable levels of 2 for skewness and 7 for kurtosis tests [69].

To test the proposed research model, we apply a two-step assessment process using SmartPLS: measurement model and structural model assessments [70]. The SmartPLS method simultaneously assesses the theoretical propositions and properties of the underlying measurement model. To validate the survey instrument, we performed a confirmatory factor analysis on all the constructs to assess the measurement model. We used SmartPLS (version 3.0) to test the convergent and discriminant validity. According to Gefen et al [71], convergent validity can be tested by examining the standardized factor loading, composite reliability, and average variance extracted (AVE). Table 3 shows the results of the convergent validity test. All values of composite reliabilities were more than the threshold value of 0.7, which highlighted that the reliability of the constructs was adequate [72]. According to Hair et al [73], a factor loading of 0.7 or greater is acceptable. In this study, all reported standardized factor loadings were greater than 0.7. The AVE of each construct was calculated using standardized factor loadings. All reported values of the AVE were also greater than 0.5, which met the minimum requirement [74]. These measures indicated that the convergent validity of the measurement model was acceptable. As the instrument validation results were satisfactory, the scales were not purified, and no items were excluded from further analysis. Thus, Table 3 includes all items used in the questionnaire.

**Table 3:** Results of convergent validity.

Construct and items		Standardized factor loading (>0.7)	Composite reliability (>0.7)	Average variance extracted (>0.5)
**IT^a^ identity**				
	ITI–REL1^b^	0.83	0.923	0.706
	ITI–REL2	0.80	N/A^c^	N/A
	ITI–REL3	0.87	N/A	N/A
	ITI–REL4	0.86	N/A	N/A
	ITI–REL5	0.84	N/A	N/A
	ITI–EMO1^d^	0.80	0.917	0.689
	ITI–EMO2	0.85	N/A	N/A
	ITI–EMO3	0.83	N/A	N/A
	ITI–EMO4	0.82	N/A	N/A
	ITI–EMO5	0.85	N/A	N/A
	ITI–DEP1^e^	0.86	0.920	0.698
	ITI–DEP2	0.87	N/A	N/A
	ITI–DEP3	0.76	N/A	N/A
	ITI–DEP4	0.79	N/A	N/A
	ITI–DEP5	0.89	N/A	N/A
**IT mindfulness**				
	ITM–ALT1^f^	0.79	0.870	0.690
	ITM–ALT2	0.82	N/A	N/A
	ITM–ALT3	0.88	N/A	N/A
	ITM–AW1^g^	0.80	0.869	0.690
	ITM–AW2	0.86	N/A	N/A
	ITM–AW3	0.83	N/A	N/A
	ITM–OP1^h^	0.90	0.917	0.787
	ITM–OP2	0.86	N/A	N/A
	ITM–OP3	0.90	N/A	N/A
	ITM–OR1^i^	0.80	0.836	0.629
	ITM–OR2	0.79	N/A	N/A
	ITM–OR3	0.79	N/A	N/A
**Perceived health status**				
	PHS1^j^	0.80	0.918	0.737
	PHS2	0.89	N/A	N/A
	PHS3	0.84	N/A	N/A
	PHS4	0.90	N/A	N/A
**Feature use behavior**				
	FEAT1^k^	0.86	0.895	0.681
	FEAT2	0.81	N/A	N/A
	FEAT3	0.80	N/A	N/A
	FEAT4	0.83	N/A	N/A
**Enhanced use**				
	ENH1^l^	0.83	0.914	0.727
	ENH2	0.87	N/A	N/A
	ENH3	0.85	N/A	N/A
	ENH4	0.86	N/A	N/A
**Continued intention to use**				
	CIU1^m^	0.82	0.953	0.801
	CIU2	0.92	N/A	N/A
	CIU3	0.92	N/A	N/A
	CIU4	0.88	N/A	N/A
	CIU5	0.93	N/A	N/A
**Positive word-of-mouth intention**				
	PWOM1^n^	0.82	0.881	0.712
	PWOM2	0.87	N/A	N/A
	PWOM3	0.84	N/A	N/A

^a^IT: information technology.

^b^ITI–REL: IT identity–relatedness.

^c^N/A: not applicable.

^d^ITI–EMO: IT identity–emotional energy.

^e^ITI–DEP: IT identity–dependence.

^f^ITM–ALT: IT mindfulness–alertness to distinction.

^g^ITM–AW: IT mindfulness–awareness of multiple perspectives.

^h^ITM–OP: IT mindfulness–openness to novelty.

^i^ITM–OR: IT mindfulness–orientation in the present.

^j^PHS: perceived health status.

^k^FEAT: feature use behavior.

^l^ENH: enhanced use.

^m^CIU: continued intention to use.

^n^PWOM: positive word-of-mouth intention.

We also tested the discriminant validity of the constructs (Table 4). All the diagonal values (the square roots of the AVEs) were greater than 0.7 and exceeded the correlations between any pair of constructs [75]. Therefore, the result indicates that the model fulfills the requirements of discriminant validity, and it is assumed that the model also has adequate discriminant validity.

**Table 4:** Results of discriminant validity.

Construct	Mean	SD	ITI-REL^a^	ITI-EMO^b^	ITI-DEP^c^	ITM-ALT^d^	ITM-AW^e^	ITM-OP^f^	ITM-OR^g^	PHS^h^	FEAT^i^	ENH^j^	CIU^k^	PWOM^l^
ITI-REL	3.37	1.00	*0.840* ^m^	N/A^n^	N/A	N/A	N/A	N/A	N/A	N/A	N/A	N/A	N/A	N/A
ITI-EMO	3.34	1.03	0.590	*0.830* ^m^	N/A	N/A	N/A	N/A	N/A	N/A	N/A	N/A	N/A	N/A
ITI-DEP	3.43	1.02	0.615	0.639	*0.835* ^m^	N/A	N/A	N/A	N/A	N/A	N/A	N/A	N/A	N/A
ITM-ALT	3.46	1.01	0.323	0.479	0.464	*0.830* ^m^	N/A	N/A	N/A	N/A	N/A	N/A	N/A	N/A
ITM-AW	3.96	0.85	0.356	0.421	0.378	0.663	*0.830* ^m^	N/A	N/A	N/A	N/A	N/A	N/A	N/A
ITM-OP	3.85	0.97	0.229	0.393	0.446	0.671	0.634	*0.887* ^m^	N/A	N/A	N/A	N/A	N/A	N/A
ITM-OR	3.76	0.87	0.315	0.373	0.399	0.546	0.618	0.526	*0.793* ^m^	N/A	N/A	N/A	N/A	N/A
PHS	3.86	1.03	0.143	0.123	0.122	0.168	0.284	0.198	0.184	*0.858* ^m^	N/A	N/A	N/A	N/A
FEAT	3.89	0.83	0.451	0.464	0.515	0.478	0.596	0.522	0.553	0.272	*0.825* ^m^	N/A	N/A	N/A
ENH	3.67	0.92	0.546	0.531	0.496	0.497	0.516	0.517	0.485	0.270	0.523	*0.852* ^m^	N/A	N/A
CIU	4.25	0.84	0.244	0.257	0.322	0.271	0.523	0.453	0.454	0.401	0.545	0.511	*0.89* ^m^	N/A
PWOM	4.00	0.84	0.451	0.449	0.457	0.417	0.544	0.570	0.490	0.336	0.561	0.547	0.559	*0.843* ^m^

^a^ITI-REL: IT identity–relatedness.

^b^ITI-EMO: IT identity–emotional energy.

^c^ITI-DEP: IT identity–dependence.

^d^ITM-ALT: IT mindfulness–alertness to distinction.

^e^ITM-AW: IT mindfulness–awareness of multiple perspectives.

^f^ITM-OP: IT mindfulness–openness to novelty.

^g^ITM-OR: IT mindfulness–orientation in the present.

^h^PHS: perceived health status.

^i^FEAT: feature use behavior.

^j^ENH: enhanced use.

^k^CIU: continued intention to use.

^l^PWOM: positive word-of-mouth intention.

^m^Greater than 0.7 and higher than the correlations between any pair of constructs.

^n^N/A: not applicable.

### Control Variables

Factors that do not represent the core variables (ie, those included in the causal model) of this study, which may affect the interrelationships between the core variables, have been controlled for. As mentioned previously, we controlled for age, gender, race, income, employment, education, the purpose of use, and experience with a PHD. Although the causal model seems to represent individuals’ active, automatic, and commitment use behaviors, we found that the effects of control variables were not negligible. On the basis of the findings, age and education influence feature use (β=−.20; *P*=.008; and β=.12, *P*=.02), which implies that younger users with higher education levels may exhibit a greater extent and breadth of use. Among the control variables, only education level influenced enhanced use (β=.19; *P*=.006). This result indicates that users with higher education backgrounds are more likely to use a formerly unused set of features for additional tasks. Age was the only control variable affecting continued intention to use (β=−.13; *P*=.03), indicating that older users are more likely to continue to use their PHDs. Finally, gender positively influences positive WOM intention (β=.18; *P*=.004). However, no effects of race, income, and purpose of use were found on any of the 4 use behaviors.

### Structural Model

**Figure 2:**
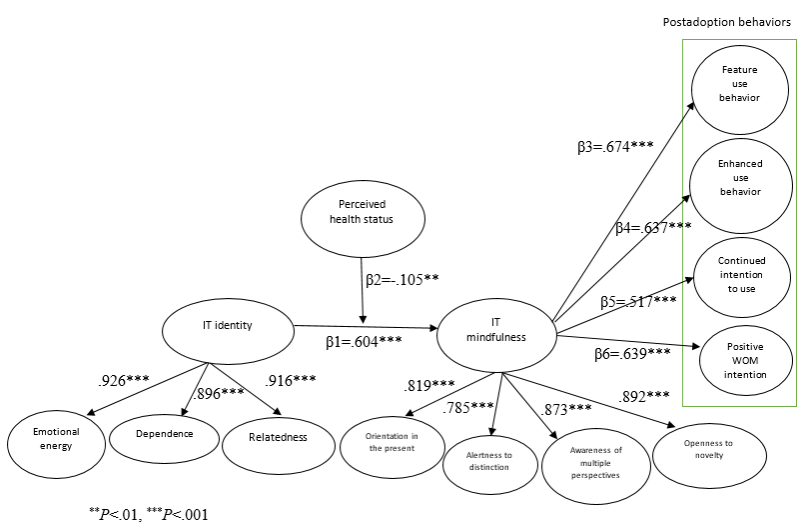
Model paths. IT: information technology; WOM: word-of-mouth.

SmartPLS (version 3.0) was used to test the hypotheses within a structural equation modeling framework. According to Ho [76], the goodness-of-fit statistics can evaluate the entire structural model and assess the overall fit. The findings indicated the normed chi-square value of 2.5, which was between the recommended values of 1 and 3 [77]. The values for indices, that is, comparative fit index of 0.92, normed fit index of 0.91, relative fit index of 0.93, and Tucker-Lewis index of 0.90, were above 0.9, and the index values for standardized root mean residual of 0.05 and root mean square error of approximation of 0.06 were below 0.08 [78]. The value of adjusted goodness-of-fit index (GFI) was 0.91, which exceeded 0.90. All these measures of fit were within the acceptable range, and only the GFI of 0.82 was marginal and could not meet the expected threshold value (which is >0.90). However, based on a study by Kline [79], at least four of the statistical values met the minimum recommended values, which supported a good fit between the hypothesized model and the observed data. Figure 2 displays the standardized path coefficients of the structural model under investigation.

In this study, IT identity comprises 3 interrelated dimensions (relatedness, emotional energy, and dependence), and IT mindfulness comprises 4 interconnected dimensions (orientation in the present, alertness to distinction, awareness of multiple perspectives, and openness to novelty). The main reason for modeling the first-order constructs as reflective constructs is the expectation of interaction among the dimensions of the second-order construct [80]. This expectation was later confirmed by the presence of significant positive correlations between the 3 dimensions (Table 4). In a reflective construct, the dimensions have positive and significant intercorrelations as they share the same pattern [81]. Moreover, the findings show that all 3 dimensions of IT identity as first-order factors load significantly on the second-order construct, as the loadings were 0.92 for emotional energy, 0.89 for dependence, and 0.91 for relatedness. Similarly, the 4 dimensions of IT mindfulness as first-order factors load significantly on the second-order construct, as the loadings were 0.81 for orientation in the present, 0.78 for alertness to distinction, 0.87 for awareness of multiple perspectives, and 0.89 for openness to novelty. Thus, the combination of 4 dimensions reflects IT mindfulness in relation to PHDs. These characteristics are more indicative of a reflective construct.

To perform the partial least squares (PLS) structural equation modeling analysis, we determined one particular indicator per construct as a dominant indicator that correlates positively with the construct [82]. This approach avoids the issue of sign indeterminacy in PLS path modeling. The structural model was assessed by examining the path coefficients. We used bootstrapping to determine the significance of each path through *t* tests. The results of the hypotheses testing are summarized in Table 5. The findings support H1 by showing a significant positive relationship between IT identity and IT mindfulness (β=.604; *P*<.001). We analyzed the interaction terms to examine whether perceived health status moderates the impact of IT identity on IT mindfulness. One path goes from the interaction term to IT mindfulness; this path tests whether perceived health status moderates the relationship between IT identity and IT mindfulness (H2). To examine the moderating effect, we used the product indicator approach. As recommended by Henseler and Chin [83], when the exogenous or the moderator variable or both are formative constructs, the two-stage PLS approach for estimating moderating effects is a better approach than the product indicator approach. In this study, as none of the exogenous or moderator variables is formative, the product indicator method is preferred. Moreover, the product indicator approach is suggested to be easily implementable in PLS path modeling [84]. The product term serves as an indicator of the interaction term in the structural model. This analysis indicates that the moderation hypothesis is supported (H2: β=−.105; *P*=.003). Therefore, the path from the interaction term has significant negative beta coefficients, indicating that the relationship between IT identity and IT mindfulness is negatively moderated by perceived health status. The *t* value for perceived health status’s moderating effect was 2.623.

**Table 5:** Results of hypotheses testing.

Hypothesis	Path	Standardized coefficient	*P* value	Standard error	*t* statistics (*df*=25)	Results
H1	ITI^a^→ITM^b^	.604	<.001	0.040	15.086	Supported
H2	ITI→ITM (moderating effect of perceived health status)	−.105	.003	0.040	2.623	Supported
H3	ITM→feature use behavior	.674	<.001	0.042	16.141	Supported
H4	ITM→enhanced use	.637	<.001	0.038	16.719	Supported
H5	ITM→continued intention to use	.517	<.001	0.055	9.404	Supported
H6	ITM→positive WOM^c^ intention	.639	.007	0.041	15.442	Supported

^a^ITI: information technology identity.

^b^ITM: information technology mindfulness.

^c^WOM: word-of-mouth.

H3 is also supported where higher IT mindfulness in relation to PHDs leads to feature use behaviors (β=.674; *P*<.001). The findings provide enough evidence to support H4, which indicates that IT mindfulness significantly reinforces enhanced use behaviors (β=.637; *P*<.001). The analysis also demonstrates that individuals’ mindfulness with a PHD positively influences continued intention to use behaviors (β=.517; *P*<.001), and this positive linkage supports H5. The path coefficient of the relationship between IT mindfulness associated with PHDs and positive WOM intention is significant, supporting H6 (β=.639; *P*=.007).

Finally, the variables explained 48% of the variance in IT mindfulness, 30% of the variance in continued intention to use, 51% of the variance in enhanced use behaviors, 56% of the variance in feature use behaviors, and 45% of the variance in positive WOM intention. The R^2^ scores reflect that the model provides relatively strong explanatory power to predict the variance in postadoption behaviors in the context of PHDs.

## Discussion

### Principal Findings

In this study, we adopt IT mindfulness as a theoretical lens to articulate factors affecting postadoption behaviors of PHD. We develop a research model (including determinants and outcomes) to gain a comprehensive view of the role of IT mindfulness during the postadoption usage of PHDs. Consistent with previous studies [22], the results confirm that IT mindfulness, as a domain-specific concept, can be used by IS studies to predict context-specific behaviors. By describing IT meaningfulness and explaining its relationships with active, automated, and commitment use behaviors, IS researchers can provide practitioners and developers with practical recommendations about how to advance users’ value derived from PHD and how to retain and increase potential users. The results of this study contribute to the IS research on the area of mindfulness by examining the implications of IT mindfulness for PHD user performance.

We validated the second-order conceptualization of the IT mindfulness construct and demonstrated its utility in the context of smart health devices. As shown in the *Results* section, all 4 dimensions of IT mindfulness (ie, awareness, alertness, openness to novelty, and orientation in the present conditions) strongly contribute to the operationalization of this concept. With a better understanding of IT mindfulness, PHD system designers may be in a better position to design systems that support mindful use. Moreover, from a managerial perspective, characterizing PHD features in terms of mindfulness raises questions about how this aspect of IS operations should be managed. Practitioners and PHD developers can consider the malleability of these factors to cultivate IT mindfulness and improve consequent use behaviors. For instance, developers can add features to the PHD software to make users ready to become more mindful of PHD functionality. Previous research reports that critical thinking about how things can be done is likely to predict mindfulness, and some attributes of IT, such as the use of highly specific instructions, can hinder mindfulness [28]. One suggestion to raise IT mindfulness could be paying attention to flexible software structure so that instructions do not seem coercive to users and technical issues can be detected quickly.

Mindfulness theories indicate that excessive automation and routines are not desirable [28]. Offering customizable features and using gamification techniques coupled with defining an acceptable level of challenge for performing health-related tasks are likely to increase IT mindfulness. For example, PHD vendors can design health-related games that include multiple simple and complex steps and encourage users to participate in these challenges. The use of promotional efforts, such as providing participants with opportunities to gain points and redeem rewards in exchange for active participation, can enhance users’ experience with smart devices. These features can elevate the state of users’ awareness of PHD capabilities, alertness to the device’s distinction, engagement in the immediate health-related task context, and flexibility in system use. Another suggestion is to design factors that are integrated into social media platforms [85] that may improve IT mindfulness and enhance our understanding of how individuals explore and use PHD features. This suggestion is consistent with Junglas et al [86], indicating that adding social components and socially enabled features to digital devices can enhance the use behaviors of those technologies.

### Theoretical and Practical Implications

One of the main theoretical contributions of this research is the identification of a cause of IT mindfulness. We show that IT identity is a strong antecedent of IT mindfulness. The results of this study contend that higher IT identity related to PHD will lead to stronger IT mindfulness associated with PHD use. Therefore, one practical way to enhance IT mindfulness in the context of smart devices could be by elevating users’ IT identity. For instance, PHD vendors can stimulate users’ sense of connection, levels of enthusiasm, and reliance on their PHD to increase their IT mindfulness, such as by continually introducing new features of PHDs [49]. It is valuable for practitioners to consider the dimensions of IT identity to establish specific guidelines and mechanisms to foster the IT mindfulness of PHD users.

Another theoretical implication of this research is to study the consequences of IT mindfulness and examine its effects on users’ beliefs and behaviors. The findings shed more light on the explanatory power of IT mindfulness in predicting postadoption behaviors. Our study provides empirical evidence that IT mindfulness can be a significant factor affecting postadoption PHD use. On the basis of the results, IT mindfulness can explain additional variance in active system use (ie, enhanced use and feature use behaviors) than commitment behaviors and continued system use. Continued use defines the automatic extension of current PHD use, but active system use is finding new opportunities for changing existing use behaviors. Our results show that the relationship between IT mindfulness and active use behavior is stronger than its linkage with automated system use. In line with previous studies, mindfulness can nurture active rather than passive as well as a mechanical thinking process and motivate individuals to use an IT device to its fullest potential [50]. People with a higher level of IT mindfulness may pay more attention to their current context than obligations that restrict freedom of use behaviors [28]. Therefore, they may be more likely to accommodate their PHD use based on the situations they are experiencing. PHD developers can improve users’ IT mindfulness by providing updated and well-formatted information about the features of this technology, explaining why their devices differ from others and articulating how these systems can be used for performing different health-related tasks.

Previous studies report that IT mindfulness empowers users to apply their knowledge in a flexible manner in new and unfamiliar situations [25]. In line with the literature, the results indicate that IT mindfulness enables individuals to innovate with their PHD to enhance their feature use. Therefore, IT-mindful people are expected to explore new and untested features to perform additional health-related tasks. Moreover, we show that IT mindfulness allows individuals to find new opportunities for using the current PHD features. The dimensions of IT mindfulness will increase the possibility of reaching more in-depth usage of familiar PHD features. Thus, we provide evidence that IT-mindful people have a greater tendency to use the existing features of their PHD in various situations.

This research is the first in the stream of IS use that hypothesizes a distinct impact of IT mindfulness on commitment behaviors. The findings demonstrate that IT mindfulness in the context of PHD use is a significant predictor of positive WOM intention. A possible justification is that IT-mindful individuals tend to monitor the task environment and keep abreast of new features and novel ways of using a system to perform different tasks [87]. Thus, they may not constrain themselves to current ways of using technology and will exhibit a greater likelihood of suggesting a PHD and its unique features to others. Their elevated awareness of the system’s functionalities and applications may encourage them to form a larger level of commitment to their PHDs. This finding is consistent with results from previous studies, suggesting a strong relationship between awareness and WOM [88]. These results suggest that PHD developers consider IT mindfulness notions in their marketing campaign to promote usability as well as the value of their smart devices and increase the use rate. Specific marketing strategies in PHD companies can be developed to enhance users’ state of being alert and aware of improving their affective commitment and positive WOM intention.

To highlight the health-related context of PHD usage and explore the contingent nature of the relationship between IT identity and IT mindfulness, we use perceived health status as a health factor. These findings imply that IT identity may lead to greater IT mindfulness, particularly among users with chronic physical or mental diseases. The moderating role of perceived health status demonstrates that IT mindfulness is dynamic and amenable to change through manipulation of individuals’ health status perceptions. This is in line with previous studies, suggesting that IT mindfulness is a dynamic trait; thus, it can help vendors learn how IT can facilitate agility and flexibility rather than merely assuming that IT must benefit agility [89]. Our results show that the IT identity–IT mindfulness relationship is less substantial for users who perceive themselves to be healthier. Consequently, the likelihood that they will engage in active, automated, and commitment behaviors is lower. Therefore, users of a PHD who perceive a poor health status will exhibit higher IT identity and develop further awareness and alertness about its applications and tend to engage in a more nuanced use. The possible rationale is that poor perceptions of health status may drive unhealthier individuals to attach themselves more to their PHD and become mindful users of it in hopes of receiving promising health consequences.

There is considerable interest in understanding the interplay between perceived health status and IT mindfulness. We believe that our findings can be a useful means for exploring this relationship in greater depth. Regarding the moderating role of perceived health status, the IT identity–IT mindfulness linkage, and in turn, the use behaviors of individuals are likely to vary. This makes it difficult for a single vendor to be able to generate health-related content and features that are comprehensive enough to embrace a wide range of health issues and topics. Thus, vendors need to decide the optimal scope of health-related functions and features on which their PHDs desire to focus. They can choose to offer a broader set of functions to cover a variety of health conditions or to focus on specific health issues (chronic diseases or typical ailments). Therefore, PHDs need to be relevant to the users, and developers should consider the target audience when designing their features, functions, and applications. For instance, a PHD may only offer the features and applications required to quit smoking or alcohol use. Another example would be a device that is required to monitor more severe issues such as cancer or HIV. According to the findings, we can argue that more focused devices with functions and applications devoted to a particular health situation may increase the chances of encouraging target users to exhibit beneficial use behaviors.

### Limitations and Future Studies

It should be mentioned that the study is based only on a sample of respondents drawn from the United States. Therefore, the results may not be generalizable to all users of PHDs. It is recommended that future studies consider drawing samples from wider geographical areas, including other countries. Our study used a web-based survey to recruit participants digitally. As a self-rated sample of participants on MTurk was used, there is a small chance that some individuals were not completely aware of mobile technology and formed their mental construal of the IT artifact. Therefore, we suggest that further studies use a different method to ensure that subjects are knowledgeable about PHDs. For instance, future research can recruit informed patients who are directly referred by providers using patient self-management tools. Moreover, our study used a web-based survey to recruit participants digitally, which might induce sample selection bias. Thus, we only considered individuals who could access a computer, mobile devices, and the internet to participate in the web-based survey. Future studies can use other data collection means and sampling strategies to reach out to a sample that is generalizable to a wide range of health care consumers.

This study could also serve as a starting point for design science studies in the context of individual adoption of smart devices. In addition, this study could be viewed as an opening gate for research in the design of technology and assessing how investigated factors could shape actual performance and use of technology. In this study, no specific PHD was examined, but the general concept of a PHD was studied. For instance, it would be interesting to investigate how alternative PHD brands influence IT mindfulness enactment and, in turn, affect user positive WOM intention. Moreover, as there are many forms of consumer technologies (such as smartphones, tablets, and computers) with different IT characteristics, one promising research avenue would be to explore the effects of IT mindfulness in other contexts rather than PHDs. In this study, we defined IT as a unit of technology (hardware and software), and IT identity as well as IT mindfulness were examined in the context of general PHDs. Another promising area of research is expanding this study by examining IT identity and IT mindfulness for users of mHealth apps (software app) and analyzing the plausible differences. Our results are interpreted as personal identities and attitudes, as demonstrated by the instrument used in this study. By using this instrument, we cannot explain the results as diagnostic of some neurobiological or invariant cognitive constructs to which the individuals are condemned. It would be interesting for other studies to consider this area of research.

It should be mentioned that 3 demographic factors (ie, age, gender, and education level) that directly influence outcome variables are considered as control variables in our conceptual model. These effects could be viewed as a limitation of this study, as they may have affected the results. Future research could include these factors in the model and test their direct relationships with outcome variables. In this study, we discussed, modeled, and examined a positive relationship between IT identity and IT mindfulness. However, as a prospect for future studies, we also suggest that further research can investigate the possible effects of IT mindfulness on IT identity. This study highlights the significant moderating role of perceived health status between IT identity and IT mindfulness. Future studies could expand this moderating effect. For instance, additional research with a new study design is required to address what dimensions of IT identity could play a more significant role in shaping IT mindfulness in light of perceived health status effects. Future research should also compare the effects of specific health status (eg, physical and mental health stability) to deeply articulate whether a lack of physical and mental health stability could exert different effects on IT identity and its relationship with IT mindfulness. Furthermore, the results indicate that together, the factors were able to explain 48% of the variance in IT mindfulness. Although we controlled for confounding variables through randomization, we need to acknowledge the possible confounding effects of age, gender, education level, technology experience, and employment status in the proposed model. Another research avenue to consider is examining additional factors that may enhance the amount of variance in IT mindfulness explained (eg, trust in smart devices, personal innovativeness in IT, and computer self-efficacy).

### Conclusions

IT mindfulness is a relatively new concept in IS research. This study contributes to IS research by validating the concept of IT mindfulness as a second-order construct with 4 reflective dimensions. We also develop a research model to examine the antecedents and implications of IT mindfulness for user performance in the context of PHDs. Through an empirical study, we offer evidence to highlight the importance of the IT mindfulness construct for studying individuals’ resultant adoption behaviors within the domain of wearable health devices. Our results suggest that IT mindfulness could be cultivated through IT identity and relate closely to postadoptive PHD use. Furthermore, we demonstrate that perceived health status negatively moderates the relationship between IT identity and IT mindfulness associated with PHDs. Thus, we suggest that the link between IT identity and IT mindfulness is stronger for individuals who perceive themselves as unhealthier. The findings of this study provide insights into the phenomenon of IT mindfulness formation and add to the literature on IT mindfulness, eHealth, mHealth, self-management tools, and health informatics. Owing to the impact of IT mindfulness on postadoption behaviors, its 4 dimensions could be used for designing PHD technologies. Moreover, vendors may need to put their efforts into means of increasing IT mindfulness by reinforcing IT identity to serve and retain a wide range of target users. Theoretical and practical contributions of this study are noticeable because they could result in a deeper understanding of human beings in relation to IT systems in an evolving digital world.

## Appendix

Multimedia Appendix 1 Online survey.

## Abbreviations

AVE: average variance extracted
GFI: goodness-of-fit index
IS: information system
IT: information technology
mHealth: mobile health
MTurk: Mechanical Turk
PHD: personal health device
PLS: partial least squares
WOM: word-of-mouth
