# Supplementary material for: The Role of Information Technology Mindfulness in the Postadoption Stage of Using Personal Health Devices: Cross-Sectional Questionnaire Study in Mobile Health
Source: JMIR Mhealth Uhealth. 2020 Oct 5;8(10):e18122. doi: 10.2196/18122 (PMC7573701; doi:10.2196/18122)
Supplement: Multimedia Appendix 1 [file mhealth_v8i10e18122_app1.docx]

**Appendix – Online survey**

| Construct | Subdimensions | Items | Questions |
| --- | --- | --- | --- |
| IT identity | Relatedness  Emotional Energy  Dependence | REL1  REL2  REL3  REL4  REL5  EMO1  EMO2  EMO3  EMO4  EMO5  DEP1  DEP2  DEP3  DEP4  DEP5 | I express feelings of connectedness when thinking of myself in relation to the Personal Health device  Thinking of myself in relation to the Personal Health Device, I feel that I am close with the device  Thinking of myself in relation to the Personal Health Device, I feel a strong sense of connection with the device  Thinking of myself in relation to the Personal Health Device, I see myself linked with the device  Overall, thinking of myself in relation to the Personal Health Device, I feel that my relatedness to the device to manage my health is high  Thinking of myself in relation to the Personal Health Device, I feel an emotional attachment to the device  Thinking of myself in relation to the Personal Health Device, I feel enduring enthusiasm about the device  Thinking of myself in relation to the Personal Health Device, I express feelings of confidence in the device  Thinking of myself in relation to the Personal Health Device, I express feelings of energy  Overall, thinking of myself in relation to the Personal Health Device, I feel that my emotional energy levels to the device are high  I express feelings of reliance when thinking of myself in relation to the Personal Health Device  Thinking of myself in relation to the Personal Health Device, I feel a sense of dependence upon the device to manage my healthcare  Thinking of myself in relation to the Personal Health Device, I feel that I can count on the device to monitor my health information  Thinking of myself in relation to the Personal Health Device, I feel that I need the device to control my health status  Overall, thinking of myself in relation to the Personal Health Device, I feel that my sense of reliance on the device is high |
| IT mindfulness | Alertness to Distinction  Awareness of Multiple Perspectives  Openness to Novelty  Orientation in the Present | ALT1  ALT2  ALT3  AW1  AW2  AW3  OP1  OP2  OP3  OR1  OR2  OR3 | I find it easy to create new and effective ways of using my Personal Health Device.  I am very creative when using my Personal Health Device.  I make many novel contributions to my health-related tasks through the use of my Personal Health Device.  I am often open to learning new ways of using my Personal Health Device.  I have an open mind about new ways of using my Personal Health Device.  I use my Personal Health Device in many different ways to support my health-related purposes.  I like to investigate different ways of using my Personal Health Device.  I am very curious about different ways of using my Personal Health Device.  I like to figure out different ways of using my Personal Health Device.  I often notice how other people are using their Personal Health Device.  I attend to the ‘big picture’ of my health control project when using my Personal Health Device.  I get involved when using my Personal Health Device. |
| Perceived health status | N/A | PHS1  PHS2  PHS3  PHS4 | In general, I believe that the state of my health is good  I consider myself as a healthy individual  When it comes to chronic condition(s), I believe that my condition is excellent  Related to my health, I experience NO major pains and discomfort for an extended period of time |
| Feature use behavior | N/A | FEAT1  FEAT2  FEAT3  FEAT4 | I will use most of the Personal Health Device's features to control my health status  I will use different features of the Personal Health Device frequently  I will use the Personal Health Device in different situations to monitor my health information  I would think to use the Personal Health Device to manage my health information rather than alternative means |
| Enhanced use | N/A | ENH1  ENH2  ENH3  ENH4 | I will explore a formerly unused set of the Personal Health Device's features  I will use the Personal Health Device for additional tasks  I will use the feature extensions of the Personal Health Device for managing my health information  Overall, I will enhance my usage of the existing Personal Health Device |
| Continued intention to use | N/A | CIU1  CIU2  CIU3  CIU4  CIU5 | I intend to continue using the Personal Health Device.  I want to continue using the Personal Health Device rather than discontinue it.  I predict I will continue using the Personal Health Device.  I plan to continue using the Personal Health Device.  The chances are high that I will continue using the Personal Health Device in the future. |
| Positive word-of-mouth intention | N/A | PWOM1  PWOM2  PWOM3 | I encourage friends and relatives to be the customers of the Personal Health Device  I say positive things about the Personal Health Device to other people.  I would recommend the Personal Health Device to someone who seeks my advice. |

ALL items were measured on a 1 = Strongly Disagree, 5 = Strongly Agree Likert scale.

At the beginning of the survey, we defined a screening question to include individuals who have used (or have been using) a PHD. The screening question was:

- This study is only designed for individuals who have used (or have been using) any mobile health devices or wearable smart tools to monitor their health status, track their fitness, or check their personal health information. Please indicate if you have used any personal health devices (PHDs), name their type, and also mention the purpose of using such tools.

Additionally, in this study, PHDs were defined as smart devices, wearable activity monitors, and intelligent bracelets that can be used for monitoring, tracking, and controlling health status.
